# Peritoneal tuberculosis pretending an acute abdomen; a case report and literature review

**DOI:** 10.1016/j.ijscr.2023.108507

**Published:** 2023-07-13

**Authors:** Javad Zebarjadi Bagherpour, Soheil Bagherian Lemraski, Alireza Haghbin Toutounchi, Hojatolah Khoshnoudi, Mohammad Aghaei, Seyed Pedram Kouchak Hosseini

**Affiliations:** a Department of General Surgery, Albourz University of Medical Sciences, Karaj, Iran; b Department of General Surgery, Artesh University of Medical Sciences, Tehran, Iran; c Department of General Surgery, Imam Hosein Medical and Educational Center, Shahid Beheshti University of Medical Sciences, Tehran, Iran

**Keywords:** Case report, Acute abdomen, Peritonitis, Peritoneal tuberculosis, Mycobacterium tuberculosis

## Abstract

**Introduction and importance:**

Peritoneal tuberculosis (PTB) is an infrequent clinical condition that frequently eludes diagnosis. The scarcity of PTB cases underscores the necessity for heightened vigilance within clinical settings to detect its presence. Notably, there has been a noticeable increase in PTB incidence in recent years. *Mycobacterium tuberculosis* is the causative agent responsible for PTB, affecting multiple gastrointestinal components such as the peritoneum and hepatobiliary system. Peritoneum is a rare site for TB with broad unspecific symptoms. It can be asymptomatic to periodic signs or mimic the positive peritonitis examinations like our case.

**Case presentation:**

We have reported a case of 19-year-old male experiencing progressive abdominal pain. The presence of generalized tenderness and guarding on physical examination prompted us to perform an urgent laparotomy due to suspicion of peritonitis. During the surgery, we observed the peritoneum exhibiting widespread nodularity, resembling a disseminated seeding pattern, along with mild ascites, which raised our suspicion of peritoneal tuberculosis. Subsequently, cytological analysis of the ascitic fluid and histopathological examination of the lesions confirmed our diagnosis of peritoneal TB.

**Clinical discussion:**

We have shared our experience in facing PTB and reviewed recent papers to find further relevant information. The common presentations, probable causes, the role of imaging in diagnosis and the management are discussed for better management strategy and the best surgical decision.

**Conclusion:**

Peritoneal tuberculosis is a rare condition with challenging diagnosis. Key symptoms include vomiting, abdominal pain, ascites, weight loss, and fever. Prompt recognition and treatment are vital for better outcomes.

## Highlights

• Peritoneal tuberculosis (PTB) is a rare clinical condition that requires a high index of suspicion for diagnosis.
• The symptoms of PTB can resemble those of other abdominal diseases, making it challenging to distinguish.
• Prompt recognition and treatment initiation are crucial to prevent diagnostic delays and reduce associated morbidity and mortality.
• Some of symptoms as defuse abdominal pain, weight loss, vomiting and fever are more frequent and it should be considered as a possibility in patients which have more than one of them.

## 1 Introduction

Peritoneal tuberculosis (PTB) is a relatively rare condition that often goes undiagnosed. It requires a high level of suspicion in clinical practice to identify cases. In recent years, there has been an increase in the incidence of PTB [1]. It can affect different parts of the gastrointestinal tract, including the peritoneum and hepatobiliary system, and is caused by *Mycobacterium tuberculosis* [1]. PTB primarily affects individuals under 40 years of age and is more common in females [2].

The clinical symptoms of PTB are non-specific and can resemble other abdominal disorders such as inflammatory bowel disease, advanced ovarian cancer, deep mycosis, Yersinia infection, amebomas or a peritonitis [2]. The presentation of PTB can be acute or characterized by intermittent symptoms. The majority of patients (80–95 %) experience abdominal pain, 40–90 % develop a fever, 11–20 % may have diarrhea or constipation, and 40–90 % experience weight loss, loss of appetite, and fatigue [3]. Patients with peritoneal TB typically have gradually progressive abdominal swelling due to ascites and abdominal pain. Adhesions can also lead to small intestine obstruction. Physical examination findings may include diffuse abdominal tenderness, a soft abdomen, enlarged liver, and the presence of ascites. Risk factors associated with peritoneal TB include HIV infection, cirrhosis, diabetes, malignancy, and continuous ambulatory peritoneal dialysis [4,5]. Peritoneal TB can manifest in three different types:

1. Wet type: characterized by the presence of ascites.
2. Encysted type: presenting with abdominal swelling.
3. Fibrotic type: characterized by the formation of abdominal masses due to the thickening of the mesentery and omentum. It is also possible for a combination of these types to occur [3,5].

During the infection, the peritoneum becomes thickened, hyperemic, and loses its normal shine. Both the visceral and parietal peritoneal layers show multiple tuberculous nodules [4]. Ascites develops as a result of proteinaceous fluid exudates from these nodules. Approximately 90 % of patients with TB peritonitis experience ascites [5]. Peritoneal involvement can occur through direct extension from infected lymph nodes, intestinal lesions, or fallopian tubes in women. Additionally, abdominal lymph nodal and peritoneal TB can develop without gastrointestinal involvement in about 30 % of cases [4].

Peritoneal tuberculosis is a significant cause of ascites in developing countries [6]. It is closely associated with poor economic and sanitation conditions. Diagnosis can be made through direct biopsy using laparoscopic or laparotomy techniques, or through indirect tests such as PPD, PCR, culture, or Ziehl-Neelsen acid-fast staining [6,7]. In this report, we present a case of a young man who presented with complaint of worsening abdominal pain. This article has been reported in line with the SCARE 2020 criteria [13].

## 2 Presentation of case

### 2.1 History

A 19-year-old male sought medical attention at the emergency department due to extreme abdominal pain. The patient had been experiencing chronic abdominal pain for the past two months. However, over the course of the last day, he developed progressively worsening pre-umbilical pain that eventually became generalized. In addition to the pain, the patient reported feeling nauseous and had vomited twice, accompanied by a loss of appetite. He denied having any significant past medical history or previous surgical interventions.

### 2.2 Assessment

**Fig. 1:**
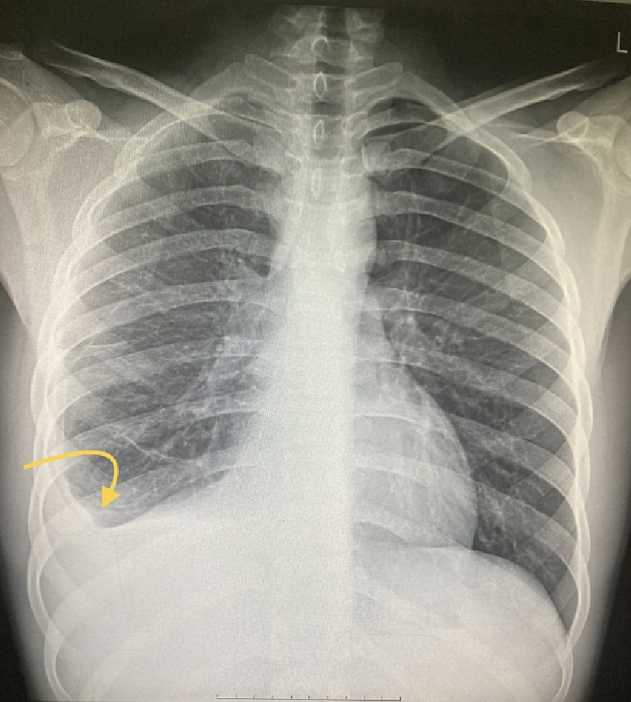
The chest X-ray.

**Figs. 2, 3:**
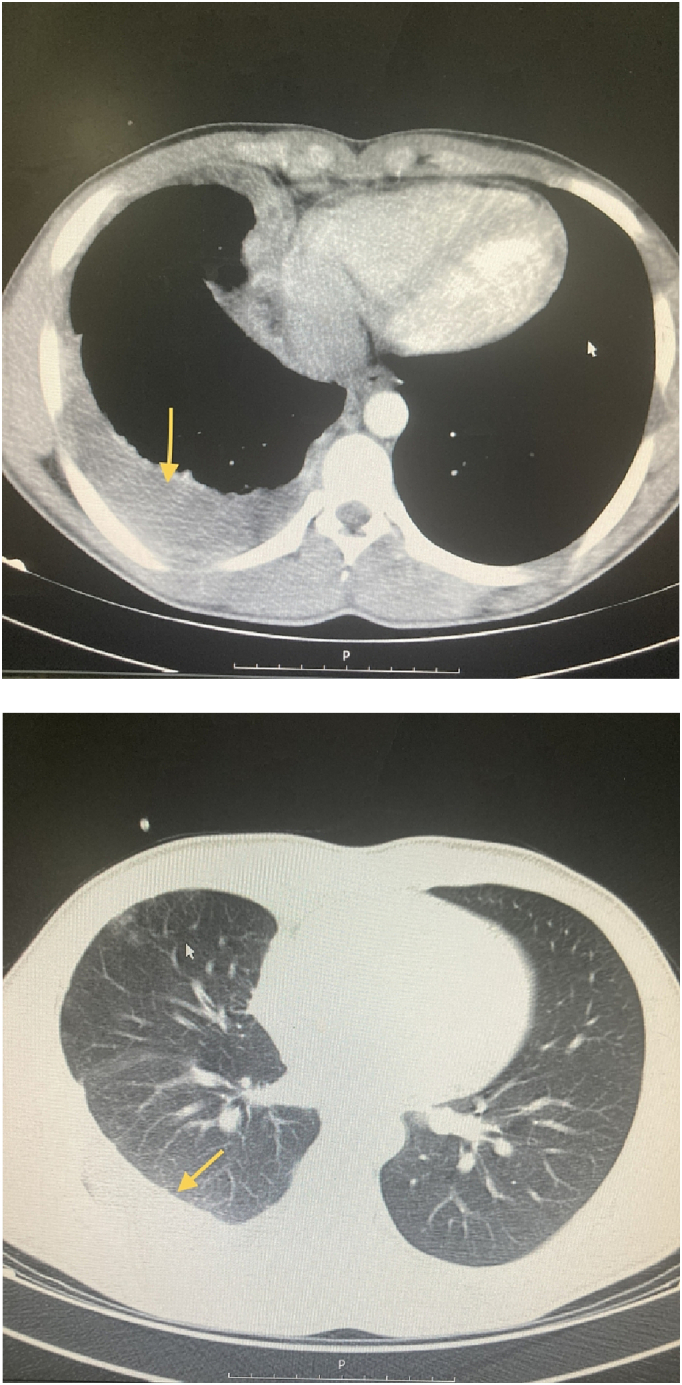
Chest CT just shows the plural effusion in right hemithorax.

**Fig. 4:**
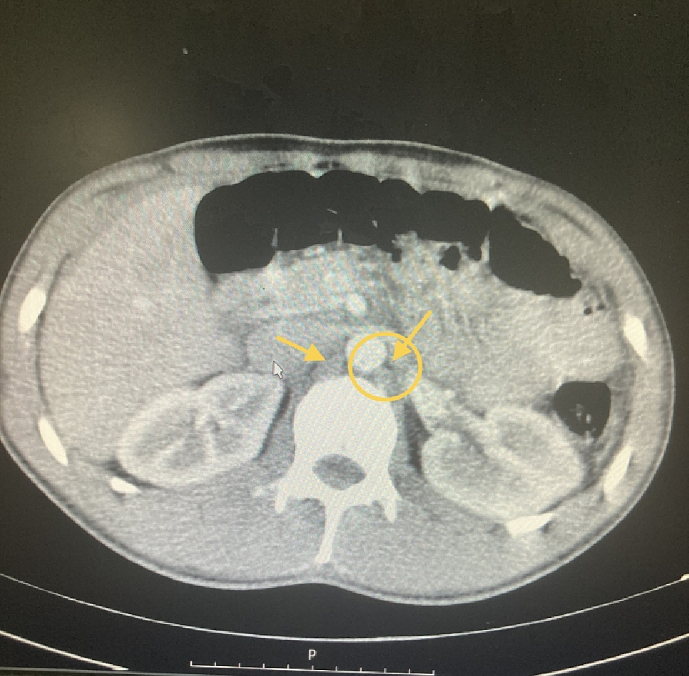
Abdominal CT shows the para-aortic lymph nodes.

Upon examination, the patient presented with generalized tenderness and guarding, without distention and rebound tenderness, and the abdomen appeared smooth. A digital rectal examination (DRE) revealed the presence of fecal stain. The patient's bowel habit history was reported as normal. Vital signs were within the normal range, and there was no indication of fever. Laboratory results showed a white blood cell count (WBC) of 25,000 and an erythrocyte sedimentation rate (ESR) of 95, other lab results were normal and viral markers tested negative. Chest X-ray findings revealed evidence of pleural effusion in the right hemithorax [Fig. 1]. Abdominal radiography was normal. Abdominopelvic ultrasound revealed the presence of free fluids and a few enlarged lymph nodes in the left lower quadrant, with a maximum short axis diameter (SAD) of 6 mm, along with decreased peristaltic activity. Subsequently, a contrast-enhanced CT scan of the thorax and abdomen demonstrated a pleural effusion in the right hemithorax, ground glass opacity, and a subphrenic mass measuring 14 ∗ 8 mm, as well as several para-aortic and mesenteric lymph nodes, and abdominopelvic free fluid in the peritoneal cavity [Figs. 2, 3, 4]. Attempted tapping of the pleural effusion was tried but unsuccessful due to loculation. However, due to the presence of peritonitis, the patient underwent a laparotomy procedure.

### 2.3 Operation summary

**Fig. 5:**
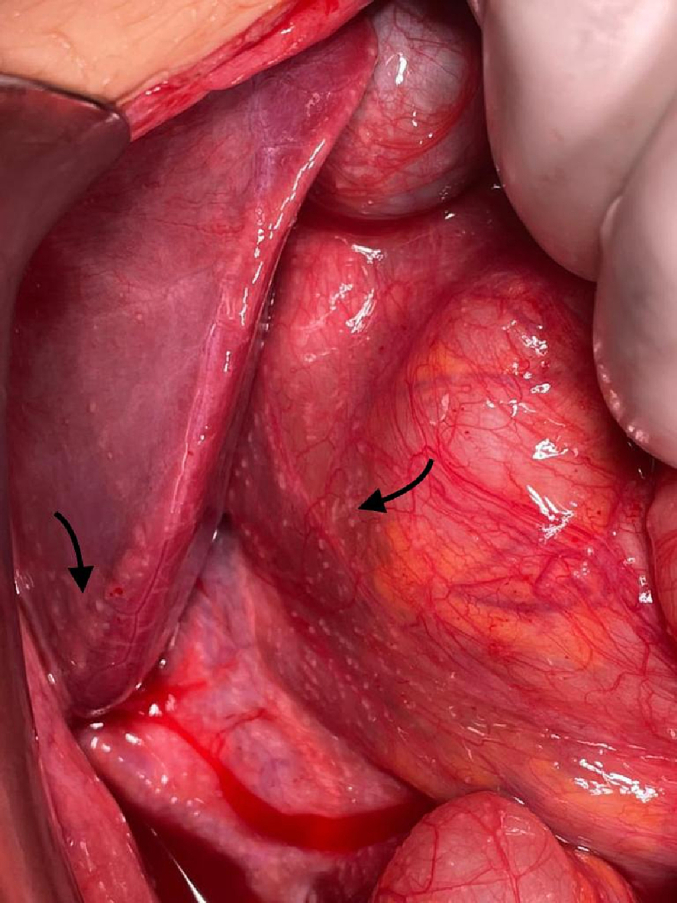
Peritoneal and liver granulomatous seeding.

**Fig. 6:**
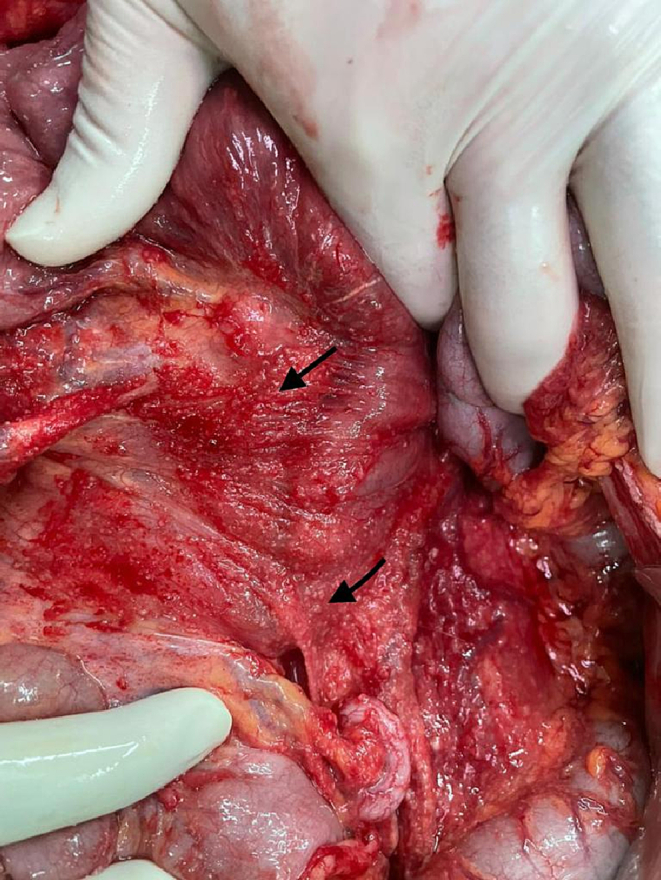
Disseminated nodular pattern of PTB on mesentery.

The laparotomy was conducted under general anesthesia via a mid-line incision, with the involvement of an attending general surgeon and a senior resident. During the exploration of the abdominal cavity, disseminated seeding lesions suspected to be related to tuberculosis (TB) were observed on the peritoneum and mesentery of the intestines, along with two larger masses on the left lobe of the liver [Fig. 5, Fig. 6]. Biopsy samples were obtained from the mesenteric and liver lesions for pathology, and reactive fluids were aspirated for cytological examination and bacterial culture. A comprehensive investigation of the peritoneal cavity was carried out, revealing no additional pathological defects or sources of perforation, which were subsequently closed. Postoperatively, broad-spectrum intravenous antibiotics were administered due to unknown probable source of infection until the result of culture be ready.

### 2.4 Outcome

Pathology reported severe chronic granulomatous inflammation for specimens and negative results for malignancy, and the fluid was positive for TB. So, treatment of TB was added for the patient with combination therapy of Rifampin/Isoniazid/Pyrazinamide/Ethambutol. He was discharged in good and stable condition with no abdominal pain in second week.

## 3 Discussion

The diagnosis of extra-pulmonary tuberculosis (TB), especially the peritoneal type, presents challenges due to the absence of specific symptoms and physical findings, particularly when there is no history or evidence of concurrent pulmonary lesions [5]. Delayed diagnosis and treatment initiation can result in increased morbidity and mortality rates [4]. When patients present with nonspecific clinical symptoms such as vomiting, abdominal pain, ascites, weight loss, and fever, PTB should be considered in the initial list of potential diagnoses [6].

Peritoneal TB is a subacute disease, and its symptoms gradually evolve over several weeks to months. Atypical presentations may occur in patients with comorbid conditions such as cirrhosis, leading to delayed diagnosis. In elderly patients, minimal constitutional symptoms may be observed, further contributing to diagnostic delays. The disease can manifest in three forms: wet-ascitic, fibrotic-fixed, and dry-plastic [4,6]. These forms share similar clinical manifestations, except abdominal distension and ascites are absent in the dry-plastic form. However, there is often an overlap between the forms, and more than one form can coexist. Systemic and constitutional symptoms are commonly observed. Low-grade fever occurs in approximately 59 % of cases and is often accompanied by night sweats. It's important to note that some patients may not report fever, and it may only be documented during hospitalization in around 49 % of cases [4,6]. Other systemic features of the disease include weight loss, anorexia, and malaise. In patients with peritoneal TB and other chronic conditions such as uremia, cirrhosis, or AIDS, quantifying these symptoms becomes even more challenging. Weight loss is observed in approximately 61 % of cases, and its reversibility has been reported as a marker of disease resolution [6,7].

**Fig. 7:**
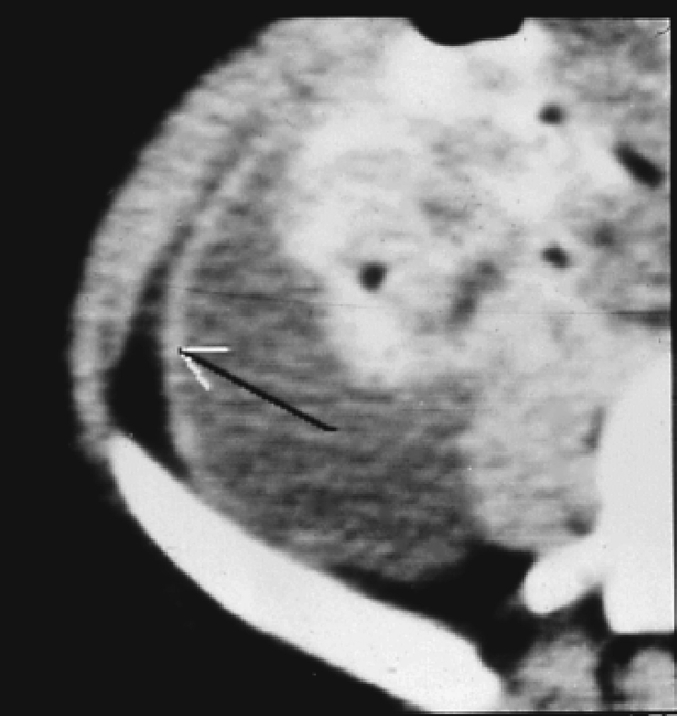
Thickened peritoneum (arrow).

Paraclinical studies can play a crucial role in supporting the diagnosis. Laboratory investigations, including hematology and ascitic fluid analysis, provide valuable information. Microbiological studies, DNA polymerase tests, and tuberculin skin tests are also available and useful in the diagnostic process. Chest X-rays show abnormalities only in 19–83 % of peritoneal TB cases, with an average of approximately 38 % based on cumulative data from over 1000 patients [6]. Active concomitant pulmonary disease is observed in only 14 % of patients. While peritoneal TB represents one of the disseminated forms of TB, miliary shadowing is rarely observed or reported in the literature [7]. Ultrasound (US) changes may include the presence of echogenic debris, fine mobile strands, or particulate matter within the ascitic fluid. Calcifications in the walls of encysted ascitic fluid are rare, but when present, they can be detected through US imaging [6]. On computerized tomographic (CT) imaging, ascitic fluid exhibits high attenuation values [8]. The peritoneum is often thickened and shows nodularity [8] [Fig. 7]. The fibrotic-fixed type of peritoneal TB is characterized by a hypervascular peritoneum, matting of the loops, and the presence of omental masses. CT scans are more effective in identifying omental changes, which occur in 36–82 % of cases [8,9]. Thickened mesentery (>15 mm) with mesenteric lymph nodes is a common finding and may serve as an early sign of abdominal TB [9]. CT scans also provide better visualization of the loss of normal mesenteric configuration. However, several studies have compared the findings of US and CT in peritoneal TB, and they have found that these imaging modalities complement each other by providing different information. US is superior to CT in revealing the multiple fine, mobile septations typically seen in peritoneal TB, while CT can better highlight the involvement of the peritoneum, mesentery, or omentum [[8], [9], [10], [11]].

The management of peritoneal TB involves various components, including a clinical approach, antituberculosis therapy, and surgical interventions. Due to the challenges in establishing a definitive diagnosis of abdominal TB, a high level of suspicion is crucial in guiding treatment decisions. Overall, although there is no specific sign or finding for definite diagnosis of PTB except the examination of direct specimen but some of symptoms as defuse abdominal pain, weight loss, vomiting and fever are more frequent and PTB should be considered as a possibility in patients which have more than one of these. Most frequent radiological finding in PTB is ascitic fluid and debris and characteristics of bacterial ascites in US sonography, and peritoneal thickening in CT scan, but as it said above CXR can be normal in most of cases.

However, in such cases pretending symptoms of an acute abdomen, we could choose to close observe if the surgeon is not convinced enough about peritonitis or doing diagnostic invasive procedure as laparoscopy or laparotomy. The best optimal plane in this scenario always is doing a diagnostic laparoscopy. In present case we did laparotomy according to evidence and positive examination of peritonitis dou to our center urgent facilities.

In stablished PTB cases, the administration of antituberculous treatment, similar to that of pulmonary TB, becomes essential. The standard therapeutic regimen typically consists of daily doses of Isoniazid, Rifampin, Pyrazinamide, and Ethambutol. However, in complicated cases, such as those involving obstruction, invasive interventions and surgical procedures may be necessary [12]. Further research is needed to enhance our understanding of diagnostic strategies, optimal treatment regimens, and long-term management of peritoneal tuberculosis.

## 4 Conclusion

Peritoneal tuberculosis (PTB) is a rare clinical condition that requires a high level of suspicion for accurate diagnosis. It is important to promptly recognize and initiate treatment to avoid delays in diagnosis and reduce the associated risks. The symptoms of PTB are unspecific, but certain symptoms such as vomiting, abdominal pain, ascites, weight loss, and fever should raise concerns about PTB. The most frequent radiological finding in PTB is ascitic fluid and debris and characteristics of bacterial ascites in US sonography, and peritoneal thickening in CT scan, but as said above CXR can be normal in most cases. Early recognition and timely initiation of treatment are essential for improved patient outcomes.

## Consent

Written informed consent was obtained from the patient for publication of this case report and accompanying images. A copy of the written consent is available for review by the Editor-in-Chief of this journal on request.

## Ethical approval

Ethical approval is exempt/waived at our institution for case reports.

Shahid Beheshti University of Medical Sciences.

## Funding

This article did not receive fund.

## Author contribution

Dr. Javad Zebarjadi Bagherpour is the main author and he has designed this report.

Dr. Soheil Bagherian Lemraski participated in Conceptualization.

Dr. Alireza Haghbin Toutounchi participated in writing and Methodology.

Dr. Mohammad Aghaei participated in Investigation and Term.

Dr. Hojatolah Khoshnoudi participated in Data curation.

Dr. Seyed Pedram Kouchak Hosseini is the writer of this article and corresponding author.

## Guarantor

Dr. Javad Zebarjadi Bagherpour accepts all responsibility of this article.

## Research registration number

Not applicable.

## Declaration of competing interest

All authors declare that they have no conflicts of interest.
